# MGMT methylation and its prognostic significance in inoperable IDH-wildtype glioblastoma: the MGMT-GBM study

**DOI:** 10.1007/s00701-024-06300-x

**Published:** 2024-10-05

**Authors:** Prajwal Ghimire, Ahmad Kamaludin, Berta F. Palau, Jose P. Lavrador, Richard Gullan, Francesco Vergani, Ranjeev Bhangoo, Keyoumars Ashkan

**Affiliations:** 1 School of Biomedical Engineering & Imaging Sciences, King’s College London, London, UK; 2 Department of Neurosurgery, King’s College Hospital, London, UK

**Keywords:** MGMT, IDH-wildtype glioblastoma, Survival, Predictive biomarker

## Abstract

**Introduction:**

The methylation of the O6-Methylguanine-DNA Methyltransferase (MGMT) promoter is a valid biomarker for predicting response to therapy with alkylating agents and, independently, prognosis in IDH-wildtype(IDH-w) glioblastoma. We aim to study the impact of its methylation in overall survival of the unresectable IDH-w glioblastoma undergoing biopsy and systemic treatment.

**Methods:**

We collected six-year retrospective (2017–2023) data at a quaternary neurosurgery center for patients undergoing biopsy as the only surgical procedure for an unresectable IDH wildtype glioblastoma. Data was collected from patient records including neuro-oncology multidisciplinary team meeting (MDT) documentation. Patients were grouped into categories according to different types of treatment received after biopsy (no treatment, chemotherapy (CT), radiotherapy (RT), chemoradiotherapy (CRT), chemoradiotherapy with adjuvant temozolomide (CRT with adjuvant TMZ), EORTC-NCIC protocol followed by second line treatment) and according to methylation status (unmethylated (< 5%), borderline methylated (5–15%) and strongly methylated (> 15%)). Survival analysis was performed.

**Results:**

166 glioblastoma IDH wildtype patients were included in the study with mean age of 62.5 years (M: F = 1.5: 1). 70 (49.3%) patients had unmethylated MGMT status (< 5%), 29 (20.4%) patients had borderline methylated MGMT status (5–15%) and 43 (30.2%) patients had methylated MGMT status (> 15%). 36 (25.3%) patients did not receive any treatment post biopsy, 13 (9.1%) received CT only, 27 (19%) RT only, 12 (8.4%) CRT, 33 (23.2%) CRT with adjuvant TMZ, whereas 21 (14.7%) received EORTC-NCIC protocol along with second line treatment.

In biopsy only group, there was no notable difference in survival outcomes among the different methylation statuses. For biopsy and any-other-form-of-treatment methylated groups showed a distinct trend of better survival compared to the borderline or unmethylated groups. Overall, methylated patients had better survival as compared to unmethylated or borderline groups.

**Conclusion:**

Methylated MGMT status are predictors for better overall survival in unresectable IDH wildtype glioblastoma patients undergoing biopsy and treatment regardless of the treatment modality.

## Introduction

O6-methylguanine-DNA methyltransferase (MGMT) promoter methylation is shown to have survival benefit for glioblastoma patients undergoing maximal safe resection, radiotherapy and concomitant temozolomide (TMZ) followed by 6 cycles of adjuvant TMZ, acting as prognostic marker [8, 12]. Furthermore, MGMT promoter methylation has now been shown to act as a predictive marker for response to TMZ in newly diagnosed glioblastoma. However, there is lack of level 1 evidence for impact of MGMT methylation for inoperable glioblastoma undergoing biopsy and systemic treatment [2, 3]. The systemic treatment options currently for inoperable glioblastoma undergoing biopsy only are radiotherapy, chemotherapy, EORTC-NCIC protocol [12] and/or addition of second line chemotherapy depending on factors such as performance status, tolerance of treatment and other prognostic factors usually recommended through multidisciplinary team discussion (MDT) [2, 3, 6].

The aim of our study is to understand the impact of MGMT methylation on inoperable IDH-wildtype glioblastoma patients who undergo biopsy and/or systematic treatment.

## Materials and methods

MGMT-GBM is a retrospective single center study investigating consecutive IDH-wildtype glioblastoma patients who underwent biopsy at a quaternary neuro-oncology center. Neuro-oncology MDT and patient notes from digital hospital records were utilized to collect data from 2017 to 2023 covering a period of six years.

### Inclusion criteria

All adult patients with IDH-wildtype glioblastoma undergoing biopsy were included and clinical data along with MGMT methylation data were collected.

### Exclusion criteria

Any patient who underwent subsequent resective surgery after the biopsy were excluded. Patients whose histological diagnosis were revised to grade 4 astrocytoma were excluded even if were given a glioblastoma diagnosis at the time of biopsy.

### Patient categories

Patients were grouped into six treatment groups that included biopsy only without any subsequent systemic treatment, chemotherapy only, radiotherapy only, chemoradiotherapy, chemoradiotherapy with adjuvant temozolomide (TMZ) (EORTC-NCIC protocol), chemoradiotherapy with adjuvant TMZ and second line treatment such as lomustine, etoposide or PCV (procarbazine, lomustine and vincristine combination). These groups were further pooled by three methods:

1. EORTC-NCIC protocol: This method of pooling categorized the groups into 3: “no treatment”, “complete or incomplete EORTC-NCIC protocol” and “EORTC-NCIC protocol with second line treatment”.
2. Treatment: This method of pooling categorized the groups into 2: “No treatment” and “any treatment”. Any treatment included all the categories of systemic treatment.
3. TMZ therapy: This method of pooling categorized the groups into 3: “Any TMZ therapy”, “only RT” and “No treatment”

### MGMT methylation status categories

There were three categories for MGMT methylation [5]: MGMT 0: unmethylated (< 5%); MGMT 1: borderline methylated (5–15%) and MGMT 2: methylated (> 15%).

### Data analysis

Data were analyzed with Chi-square test, one-way ANNOVA, Kaplan Meier survival analysis, log-rank test and cox proportional hazard model using Python and Microsoft Excel 2024. *P* value of < 0.05 were considered to be statistically significant.

## Results

One hundred and sixty-six patients were identified from the patient records for the study. Two patients were identified to have a revised diagnosis of high-grade astrocytoma and were excluded. Similarly, two patients went on to have a debulking surgery following the initial procedure of biopsy and thus were excluded. Twenty patients had missing data on systemic treatment as they were referred to other oncology centers for treatment. Therefore, 142 patients were available for analysis**.** Patient demographics and clinical data are listed in Tables 1 and 2.

**Table 1:** Demographics of IDH wildtype glioblastoma patients stratified by MGMT methylation status

Variable	MGMT unmethylated (< 5%) (MGMT 0)	MGMT borderline methylated (5–15%) (MGMT 1)	MGMT methylated (> 15%) (MGMT 2)	*p*-value	Test statistic	Total
Sample Size	70 (49.30%)	29 (20.42%)	43 (30.28%)	0.0001*	18.352	142
Mean Age ± SD (years)	61.36 ± 12.24	63.83 ± 12.66	63.30 ± 9.48	0.5	0.635	62.45 ± 11.54
Gender distribution (M:F)	1.5:1	1.64: 1	1.39: 1	0.9	0.112	1.5: 1
ATRX status (lost: preserved)	2: 68	0:29	4: 39	0.1	4.342	6: 136
Overall Mean Survival ± SD (Range)(days)	229.61 ± 167.93 (29–758)	260.86 ± 247.11 (21 -1087)	331.77 ± 344.67 (36 – 1740)	0.1	2.08	266.14 ± 250.04 (21 – 1740)
Pre-operative performance status (WHO)				0.1	12.18	133
0	22 (16.5%)	15 (11.3%)	12 (9%)			49 (36.7%)
1	35 (26.3%)	7 (5.2%)	20 (15%)			62 (46.4%)
2	8 (6%)	3 (2.2%)	6 (4.5%)			17 (12.6%)
3	3 (2.2%)	0	1 (0.7%)			4 (2.8%)
4	0	1 (0.7%)	0			1 (0.7%)

(*:*p* < 0.05)

**Table 2:** Demographics of IDH wildtype glioblastoma patients stratified by type of systemic treatment

Variable	CT	RT	CRT	CRT + adjuvant TMZ	CRT + adjuvant TMZ + second line treatment	No treatment	*p*- value	Test statistic	Total
Sample Size	13 (9.15%)	27 (19.01%)	12 (8.45%)	33 (23.24%)	21 (14.79%)	36 (25.35%)	0.00067*	21.437	142
Mean Age ± SD (years)	60.38 ± 13.73	68.19 ± 6.99	62.92 ± 11.74	56.12 ± 12.09	58.38 ± 12.59	66.92 ± 8.52	0.00006*	5.874	62.45 ± 11.54
Gender distribution (M:F)	1.6: 1	1.7: 1	1.4: 1	1.2: 1	1.63:1	1.57: 1	0.9	0.583	1.5:1
ATRX status (lost: preserved)	1:12	0:27	0:12	3:30	0:21	2:34	0.4	5.121	6:136
Overall Mean Survival ± SD (Range)(days)	202.15 ± 119.21 (46.00—435.00)	198.56 ± 119.43 (71.00—491.00)	186.67 ± 101.84 (81.00—385.00)	391.75 ± 191.90 (98.00—1110.00)	619.79 ± 351.67 (239.00—1740.00)	78.33 ± 46.11 (21.00—203.00)	4.62 × 10^−19^ *	28.050	266.14 ± 250.04 (21 – 1740)
Pre-operative performance status (WHO)							0.1	27.88	133
0	3 (2.2%)	11 (8.3%)	3 (2.2%)	13 (9.7%)	7 (5.3%)	12 (9%)			49 (36.7%)
1	9 (6.7%)	12 (9%)	6 (4.5%)	13 (9.7%)	6 (4.5%)	16 (12%)			62 (46.4%)
2	0	3 (2.2%)	0	3 (2.2%)	6 (4.5%)	5 (3.7%)			17 (12.6%)
3	1 (0.7%)	0	1 (0.7%)	1 (0.7%)	1 (0.7%)	0			4 (2.8%)
4	0	0	1 (0.7%)	0	0	0			1 (0.7%)

(*: *p* < 0.05)

### Methylation status

Unmethylated MGMT (MGMT 0) patients were 70 (49.3%) as compared to borderline methylated (MGMT 1) 29 (20.42%) and strongly methylated patients (MGMT 2) being 43 (30.28%) (Table 1). Further pooling the borderline and methylated patients led to two categories namely unmethylated (MGMT 0) and methylated (MGMT 1 + MGMT 2).

### Treatment categories

Patients who underwent only biopsy without any other treatment were 36 (25.35%) whereas those who underwent chemotherapy (CT) were 13 (9.15%); radiotherapy (RT) were 27 (19.01%); chemoradiotherapy (CRT) were 12 (8.45%), chemotherapy ± radiotherapy and adjuvant temozolomide (CRT + adjuvant RMZ) were 33 (23.24%) and chemoradiotherapy and adjuvant temozolomide along with second line treatment were 21 (14.79%) (Table 2).

### Overall survival analysis

The median survival time for the overall cohort of patients was 184 days with survival probability at 1 year of 0.233 and at 2 years of 0.06. The best treatment group with highest survival probability was the group who had strongly methylated MGMT status (MGMT 2) undergoing EORTC-NCIC protocol with second line therapy with survival probability at 1 year of 1.

**Fig. 1:**
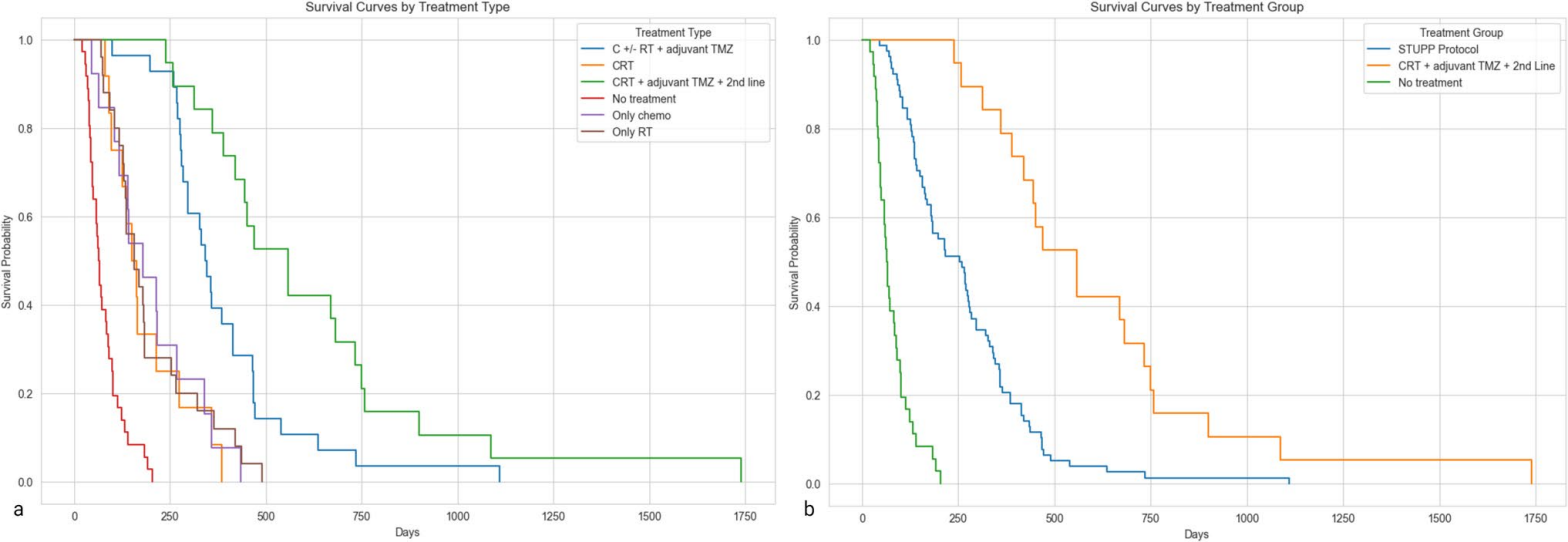
Survival curves for each treatment category. **a** Individual treatment category KM survival curves. **b** Pooled treatment category KM survival curves. There is survival benefit in patients receiving systemic therapy especially EORTC-NCIC protocol and/or second line treatment despite only undergoing biopsy. The statistical significance was observed when complete or incomplete EORTC-NCIC protocols were pooled together. (KM: Kaplan Meier, EORTC-NCIC protocol: combination of radiotherapy and concomitant temozolomide followed by 6 cycles of temozolomide; TMZ: temozolomide; RT: radiotherapy; CT: chemotherapy; CRT: chemoradiotherapy) (EORTC-NCIC protocol= STUPP protocol)

**Fig. 2:**
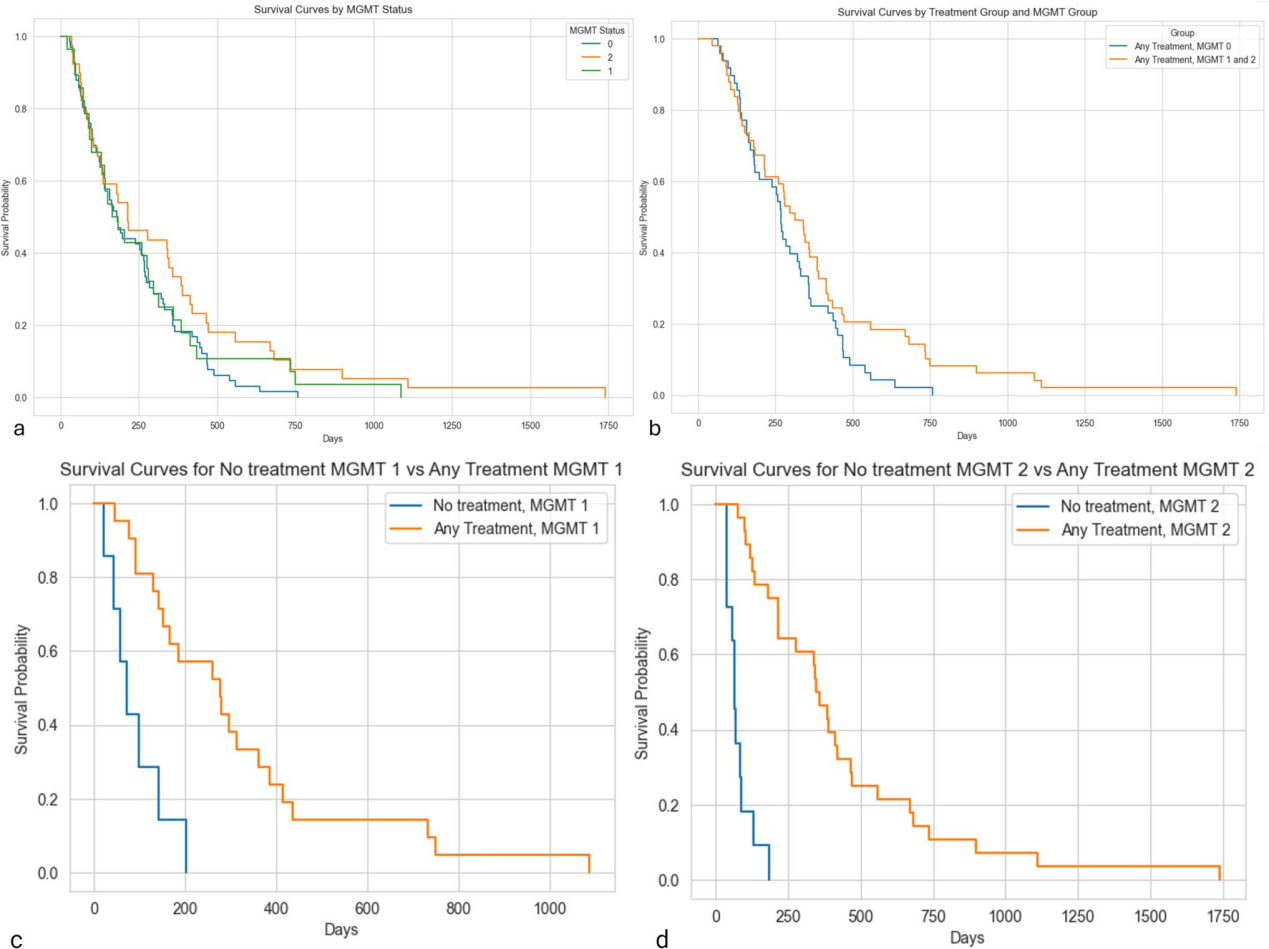
Survival curves based on MGMT status. **a** KM curve for patients in MGMT methylation categories namely unmethylated(< 5%)(MGMT 0), borderline methylated(5–15%)(MGMT 1) and strongly methylated (> 15%)(MGMT 2). **b**,**c**,**d** KM curves for the methylated MGMT categories stratified by treatment in patients who have received no systemic treatment in comparison to patients who have received systemic therapy. Survival benefit is noted in methylated groups especially with longer survival tail with any form of systemic treatment and a statistically significant difference is observed in methylated groups receiving any form of treatment as compared to no systemic treatment. (MGMT: O6-Methylguanine-DNA methyltransferase)

Regardless of the MGMT status, the survival was longest in the group who underwent EORTC-NCIC protocol along with second line treatment and lowest in patient who did not receive any systemic treatment at all (Fig. 1). Regardless of treatment strategy, the survival probability at 1 year for unmethylated status (MGMT 0) was 0.18, for borderline MGMT methylation (MGMT 1) was 0.21 and for methylated MGMT status was 0.33 (Fig. 2). Furthermore, when the treatment groups were pooled together, the survival was better in any treatment group as compared to only biopsy with no treatment group of patients (*p* = 0.005; test statistic = 113.40) (Fig. 2).

**Fig. 3:**
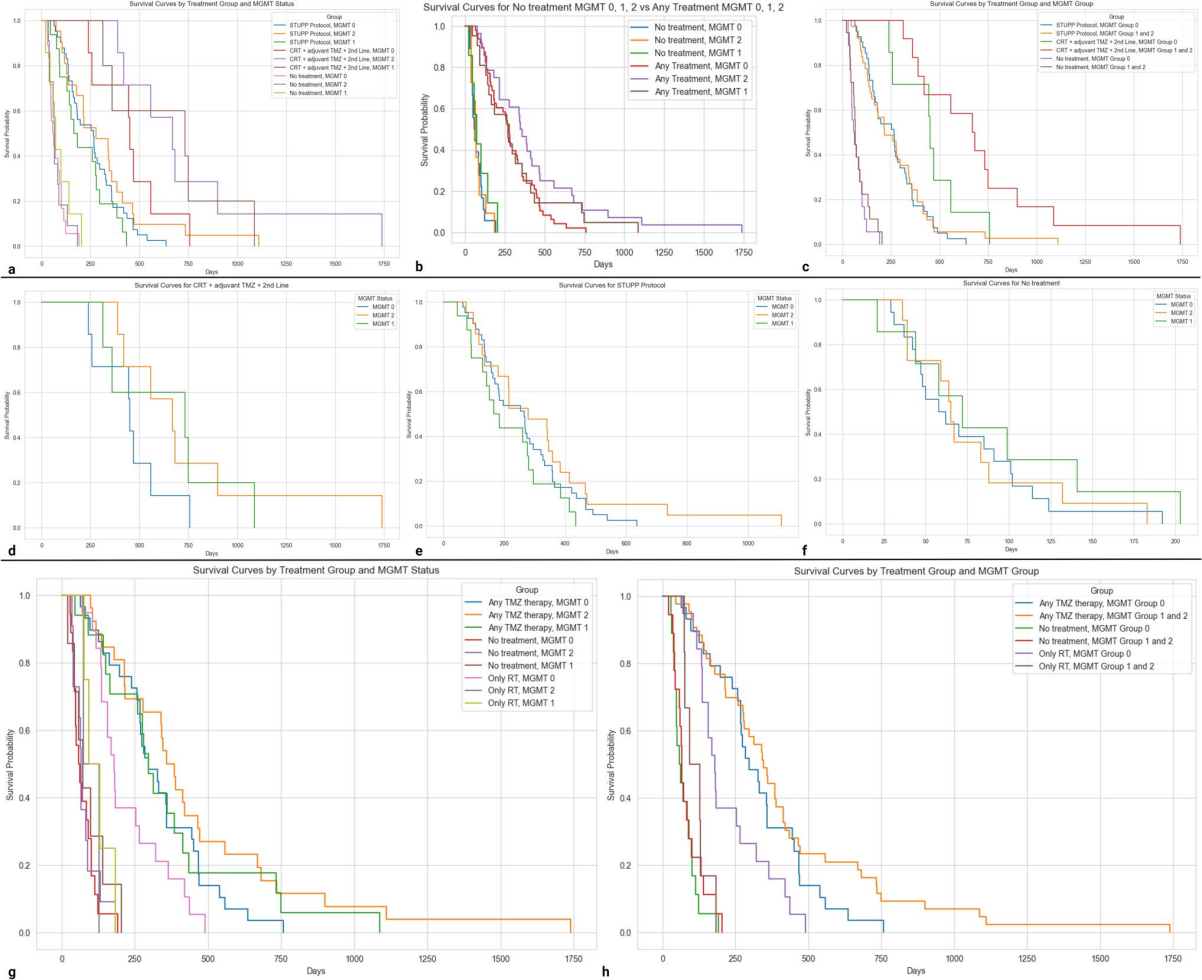
Survival curves of pooled treatment groups stratified by MGMT status. **a**,**c** Pooled treatment groups of No treatment, EORTC-NCIC protocol, EORTC-NCIC protocol and second line stratified by MGMT status. **b** Pooled treatment groups of no treatment and any treatment stratified by MGMT status. **d**,**e**,**f** Individual treatment group survival curves for EORTC-NCIC protocol, EORTC-NCIC protocol and second line treatment along with no treatment group stratified with MGMT status g,h. Pooled TMZ treatment group (Any TMZ therapy, Only RT and no treatment) survival curves stratified by MGMT status There is survival benefit in patients who receive systemic therapy in conjunction with MGMT methylation. The survival tail for strongly methylated tumors is longer for both the treatment groups that had systemic therapy. (MGMT: O6-Methylguanine-DNA methyltransferase; TMZ: temozolomide; MGMT 0: unmethylated (< 5%); MGMT 1: borderline methylated (5–15%); MGMT 2: strongly methylated (> 15%); CRT: chemoradiotherapy as a part of EORTC-NCIC protocol)

In strongly methylated patients (“MGMT 2”), treatment with EORTC-NCIC protocol followed by second line therapy had better survival outcome as compared to complete or incomplete EORTC-NCIC protocol (*p* = 0.005; test statistic = 21.92). In borderline methylated patients (“MGMT 1”), treatment with EORTC-NCIC protocol with second line therapy had better survival as compared to complete or incomplete EORTC-NCIC protocol (*p* = 0.005; test statistic = 50.29) (Fig. 3). In patients with methylated MGMT (borderline or strong; “MGMT 1” + “MGMT 2”), statistical significance for longer overall survival was observed with patients undergoing EORTC-NCIC protocol with second line therapy as compared to patients undergoing complete or incomplete EORTC-NCIC protocol (*p* = 0.0002) or patients undergoing biopsy only with no systemic therapy (*p* = 5.93e-08). Statistical significance was further observed for longer overall survival for patients undergoing complete or incomplete EORTC-NCIC protocol as compared to only biopsy with no systemic therapy (*p* = 4.047e-09). In unmethylated patients (“MGMT 0”), treatment with EORTC-NCIC protocol with second line therapy had better survival as compared to complete or incomplete EORTC-NCIC protocol (*p* = 0.005; test statistic = 84.67). Similarly, in unmethylated patients, EORTC-NCIC protocol provided patients with statistically significant survival benefit as compared to patients undergoing only biopsy with no systemic treatment (*p* = 6.838e-13). This was also true for patients receiving EORTC-NCIC protocol with second line treatment as compared to patients undergoing only biopsy with no systemic therapy (*p* = 1.686e-05) (Fig. 3).

Statistically significant longer overall survival was observed in methylated tumor patients who underwent any form of systemic treatment namely complete/incomplete EORTC-NCIC protocol and EORTC-NCIC protocol with second line treatment (*p* = 0.005; test statistic = 37.47) as compared to no treatment category along with longer survival tails (Fig. 3). In biopsy only group with no treatment, there was no notable difference in survival outcomes among the different methylated statuses (Fig. 3).

On TMZ therapy pooling, unmethylated (“MGMT 0”) group showed significant differences in survival between all treatment comparisons (any TMZ therapy, only RT and no treatment)( Any TMZ therapy vs. No treatment: *p* = 8.11e-12; Any TMZ therapy vs. Only RT: *p*-value = 0.01; No treatment vs. Only RT: *p*-value = 3.72e-07). Similarly, moderately methylated (“MGMT 1”) group showed significant differences between “Any TMZ therapy” and both “No treatment” and “Only RT”( Any TMZ therapy vs. No treatment: *p* = 4.78e-05; Any TMZ therapy vs. Only RT: *p* = 0.002). There was no significant difference between “No treatment" and “Only RT”(*p* = 0.7). In strongly methylated (“MGMT 2”) group, significant differences between “Any TMZ therapy” and both “No treatment” and “Only RT” was observed (Any TMZ therapy vs. No treatment: *p* = 1.66e-10; Any TMZ therapy vs. Only RT: *p*-value = 0.0001) with no significant differences between “No Treatment” and “Only RT”(*p* = 0.68). Overall, Methylated tumors (“MGMT 1 and 2”) showed significant differences in survival with “Any TMZ therapy” as opposed to “No treatment” or “Only RT” category(Any TMZ therapy vs. No treatment: *p* = 2.38e-14; Any TMZ therapy vs. Only RT: *p* = 3.01e-07) (Fig. 3).

### Cox proportional hazard model

**Fig. 4:**
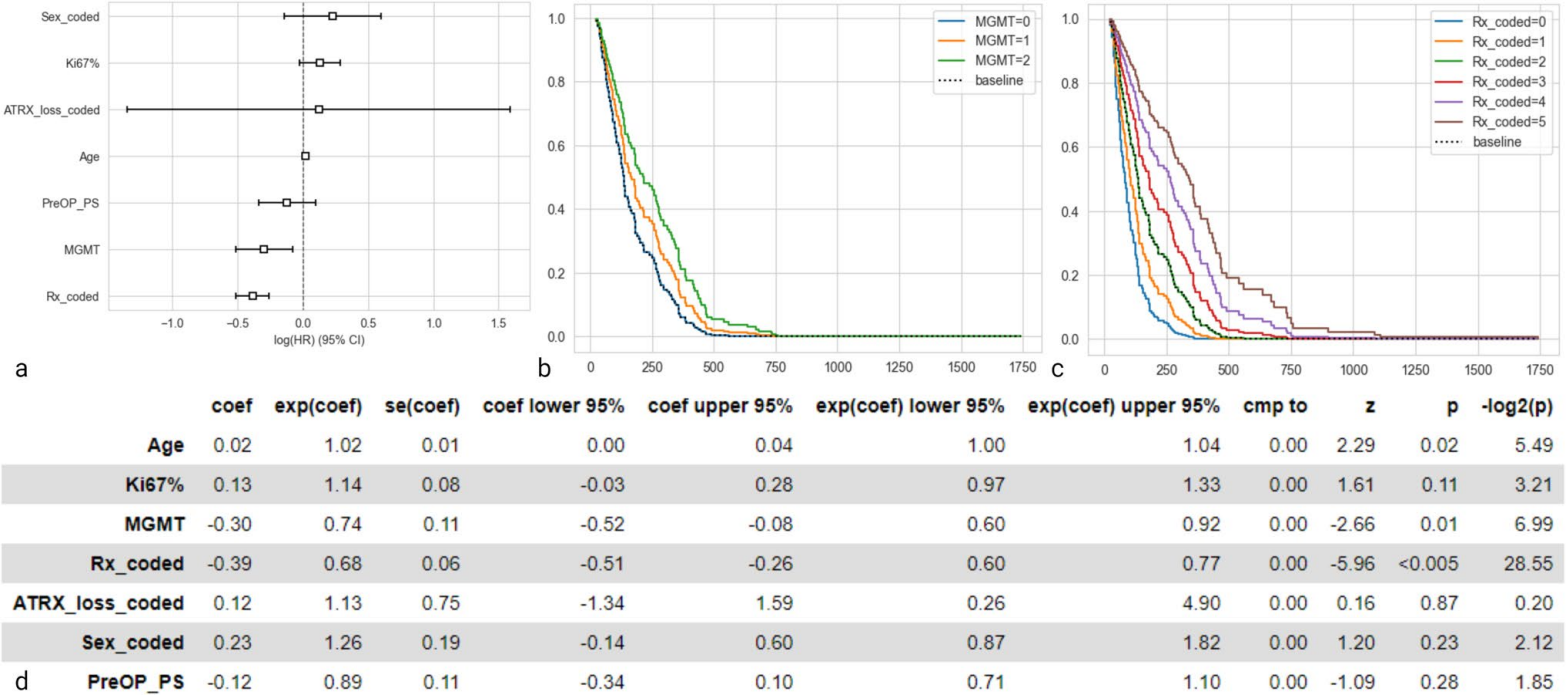
Cox proportional hazard model with reference to overall survival. There is statistically significant impact of treatment categories and MGMT methylation categories on overall survival. The model indicates statistically significant increase in hazard (shorter survival) with age; decrease in hazard (longer survival) with increasing MGMT methylation and with categories of systemic therapy. A good predictive power of the model is indicated by concordance of 0.72 and the -log2(p) value of 33.98 in the log-likelihood ratio test indicates strong evidence that the model is useful in predicting survival. **a** Coefficient plot of the model. **b** Hazard ratios of treatment categories on overall survival **c**. Hazard ratios of MGMT methylation categories on overall survival. **d** Summary of results of the hazard model (MGMT = 0: unmethylated (< 5%); MGMT = 1: borderline methylated (5–15%); MGMT = 2: strongly methylated (> 15%); Rx_coded categories are 0: no treatment, 1: CRT, 2: CRT + adjuvant TMZ, 3: only chemotherapy, 4: only radiotherapy, 5: CRT + adjuvant TMZ + second line therapy)

The survival analysis was fitted into a cox proportional hazard model with the data variables of age, WHO pre-operative performance status, Ki67%, MGMT, treatment, ATRX status and gender categories (Figure) (Fig. 4). A positive coefficient (coef = 0.02, *p* = 0.02) was observed with age indicating as the age increased, the survival of these patients decreased. The exponentiated coefficient for age was 1.02 indicating that each additional year of age increased the hazard of shorter length of overall survival by 2%. A negative coefficient (coef = -0.30; *p* = 0.01) was observed with the MGMT categories (0,1,2) indicating as the MGMT methylation increased, the length of overall survival also increased. The exponentiated coefficient for MGMT status was 0.74 indicating that each change in methylation category from unmethylated to borderline to strongly methylated was associated with a 26% lower risk of shorter overall survival (hazard decreases by a factor of 0.74). Similarly, a negative coefficient (coef = -0.39, *p* < 0.005) was observed for various treatment categories (0: no treatment, 1: CRT, 2: CRT + adjuvant TMZ, 3: only chemotherapy, 4: only radiotherapy, 5: CRT + adjuvant TMZ + second line therapy) indicating survival benefit as compared to no treatment. There was no statistical significance observed for other covariates (performance status, Ki67%, ATRX, gender). The concordance index for the model was 0.72 and the -log2(p) value was 33.98 indicating the model as a whole has statistically significant predictive power for predicting overall survival.

## Discussion

### Summary of findings

In this study, we identified that MGMT methylation impacted on the overall survival of inoperable IDH-wildtype glioblastoma undergoing systemic treatment, regardless of the treatment modality. This study is the largest sample size retrospective study in the current post-WHO-classification-2021 era for investigating the MGMT methylation impact in survival of this subgroup of patients. A higher methylation status was associated with a higher chance of longer overall survival. There were significant survival differences between patients who underwent systemic treatment with highest impact made by strong MGMT methylation (> 15%) in patients receiving EORTC-NCIC protocol along with second line treatment as compared to other treatment options and methylation statuses. There were survival benefits noted within other groups of systemic therapy too despite not achieving statistical significance in those groups, given the relatively small numbers in each group. The statistical significance was, however, observed when complete or incomplete EORTC-NCIC protocols were pooled together along with pooling of any TMZ therapy. The survival tail for strongly methylated tumors was also longer for both the treatment groups that had EORTC-NCIC protocol and/or second line treatment.

We further devised a cox proportional hazard model that predicted the overall survival of these inoperable IDH-wildtype glioblastoma based on the statistically significant predictor variables namely MGMT status and treatment categories.

### Limitations

This study, while providing valuable insights, is limited by several factors. Firstly, it is a single-center study, which may not capture the full variability of patient populations and treatment responses seen across different institutions. Secondly, its retrospective nature introduces potential biases and limits the ability to establish causal relationships between treatments and outcomes. The limited sample size further restricts the generalizability of the findings and may affect the statistical power of the conclusions drawn. To overcome these limitations, a prospective study design followed by a multicenter clinical trial is essential. This approach would facilitate the collection of high-quality, standardized data and enable a more comprehensive assessment of treatment efficacy and safety. Achieving level one evidence through such rigorous research will provide robust support for the management of patients with inoperable glioblastoma, who currently have limited standard of care treatment options.

### Current evidence in the field

There remains a dearth of studies on impact of MGMT methylation of unresectable IDH- wildtype glioblastoma patients after WHO 2021 updated classification [1]. There has been few retrospective studies prior to the classification demonstrating the impact of MGMT methylation status on overall survival of these tumors. A 2023 report from the RANO resect group [9] have identified that patients undergoing biopsy only had least favorable PFS (5 months, CI:4–6, *p* = 0.001) and OS (10 months, CI:8–12, *p* = 0.001) post receiving EORTC-NICIC protocol as compared to other resection groups. They went on to have different types of second line therapy (resection and/or other therapy, only RT, only TMZ, only lomustine, RT + TMZ or lomustine or others). They factored in the MGMT status in the univariate (unmethylated: HR:1.53, CI:1.3–1.9; *p* = 0.001) and multivariate analysis (*p* < 0.05) showing persistent significant impact on OS with worse prognosis in unmethylated tumors consistent with our results [9]. A 2011 retrospective study showed the MGMT promoter methylation was the strongest favorable predictor for overall survival in unresectable glioblastoma patients (OS, median: 20.3 vs. 7.3 months, *p* < 0.001, HR 0.30, 95% CI 0.16–0.55), and PFS (median: 15.0 vs. 6.1 months, *p* < 0.001, HR 0.31, 95% CI 0.17–0.57) and was also associated with higher frequencies of treatment response and prolonged post-recurrence survival (PRS, median: 4.5 vs. 1.4 months, *p* < 0.002, HR 0.39, 95% CI 0.21–0.71) [13]. Another study of unresectable thalamic glioblastoma with MGMT methylation showed improved survival with chemo-radiotherapy [5]. This subgroup of patients have now been addressed as a separate entity requiring focused study of treatment options [4, 10, 11]. In another single centre retrospective study, MGMT methylation status was an independent factor for hazard risk for overall survival in these patients who underwent biopsy independent of treatment and radiotherapy alone was seen to improve survival in these subgroups of patients [7].

### Implications for clinical practice

MGMT methylation acts as a predictive biomarker for overall survival in unresectable IDH-wildtype glioblastoma patients undergoing systemic therapy following a diagnostic biopsy. Tailoring systemic treatment for these methylated patients can have positive impact on survival and quality of life.

## Data Availability

No datasets were generated or analysed during the current study.
